# Effects of Black Pine Afforestation on Growth and Soil Properties at Reforested Postmine Sites in Kastamonu, Türkiye

**DOI:** 10.1002/ece3.73109

**Published:** 2026-02-17

**Authors:** Seray Özden Keleş, Osman Topaçoğlu

**Affiliations:** ^1^ Faculty of Forestry Kastamonu University Kastamonu Türkiye

**Keywords:** ecologic anatomy, natural forest, plantation, postmining restoration, reforestation success

## Abstract

Mining poses a significant threat to forest ecosystems due to its extensive degradation of vegetation, soils, and habitat structure. In recent years, however, mining companies have been legally required to rehabilitate disturbed lands and return them to their original condition once extraction activities have ceased. Reforestation thus plays a central role in restoring these degraded areas by reestablishing forest vegetation and promoting long‐term ecosystem recovery. The aim of this study was to assess how soil properties and the growth and development of *Pinus nigra* Arnold trees differ between postmining restoration forests and natural forest stands. Soil analyses revealed higher concentrations of Se, Mo, Ca, Fe, Zn, and Cu in the reforested mine sites, whereas natural *P. nigra* forests contained greater levels of P, N, and K. Tree growth measurements showed that total stem height and diameter were approximately 1.2 times greater in natural forests compared to restored mine sites, and height growth rates were also higher in natural stands. Anatomical assessments indicated no significant differences in tracheid characteristics between the two forest types, suggesting that *P. nigra* is anatomically well adapted to the conditions of restored mine lands. Overall, our findings demonstrate that both forest type and soil properties play essential roles in the establishment and development of *P. nigra* trees, and they highlight the importance of soil restoration and species selection in the success of postmining reforestation efforts.

## Graphical abstract

This paper is the first study on the mine‐land rehabilitation sites to determine the effect of Black pine plantations.

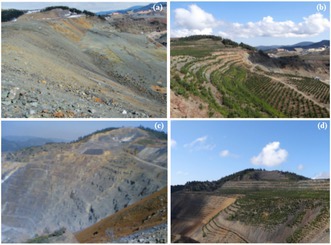

## 1 Introduction

Forest are complex ecosystems which are composed of animals, plants, trees, air, water, and soil. The degradation and loss of forests have become serious problems in many parts of the world. Mining activities are one of the biggest drivers of deforestation globally (Pritchard 1994) since the most energy and mineral resources are placed in forested regions (World Resources Institute 2014). Mining influences up to 1/3 of the forest ecosystem of the world (Hooke et al. 2012; WWF 2023) and causes serious ecological degradation such as land use change and erosion, removal of natural vegetation, soil structure, and microorganisms, and loss of biodiversity (Conrad et al. 2002; Murguía et al. 2016; Sonter et al. 2017; Kayet et al. 2021). It is important to evaluate the environmental degradations in the early stages on degraded lands. For this reason, governments have started to develop specific regulatory controls on mine closure processes (Ross 2008; H2020 MICA Project 2017; Pagouni et al. 2024). Therefore, the Environmental Impact Assessment (EIA) method has been firstly applied in the USA in the 1970s (National Environmental Policy Act) to predict the effect of mining activities on the environment; afterward, EIA applications started in European countries (Robinson 2005). In 1983, the legal EIA law provisions were enacted in Türkiye to protect and improve the environment in line with the principles of sustainable environment and sustainable development (Turkish Environment Act‐No. 2872‐Regulation 23/6/97).

Due to the severe disturbance by surface mining on forested areas, many countries established regulations or obligations to mining operations in which the mined lands should be restored and returned their original land uses during ongoing mining activities and to the end of mining (World Resources Institute 2014). Nowadays, many national‐scale mining companies therefore have been obliged to take a series of detailed environmental management strategigies depanding on the latest sustainable management plans in the forest land influenced by mining works (H2020 MICA Project 2017; Pagouni et al. 2024). The mining companies have therefore should guarantee that the degraded forest land will be returned as closely as possible to its original baseline condition after mine closure (Cooke and Johnson 2002; Wu et al. 2010; Pietrzykowski 2015a, 2015b). In recent decades, land restoration programs (i.e., forest rehabilitation, reclamation, and vegetation reestablishment treatment methods) adopted to the mined lands to restore ecological functionality, the loss of the natural vegetation, ecosystem services and biodiversity (Evans et al. 2013; Yang et al. 2018; Nie et al. 2022; Stanturf et al. 2014; Conrado da Cruz et al. 2020). The ecological restaration is determined as “the process that providing ecological integrity and recovery progress of environment which is degraded or damaged” based on the Society of Ecological Restoration. In postmining sites, main restoration practices are conservation or reconstruction of soil cover, reestablishing nutrient stock, recovery of original vegetation type and mining waste disposal.

Tree plantation‐related activities (vegetation reestablishment or reforestation) have been one of the popular practices of the rehabilitation and recovery of degraded lands influenced by mining because the general purpose of afforestation activities carried out on mine sites is to rehabilitate the land and bring it to a state equal to or better than its original state, and to carry out planting activities with species composition and diversity similar to natural ecosystems (Angel et al. 2008; Zipper et al. 2011, 2013; Skousen and Zipper 2014; Macdonald et al. 2015). In reforestation activities, the historic trajectory of mining lands before the degradation should be well known to gain higher success in vegetation reestablishment (Adams et al. 2017). The selection of native tree species and understory vegetation plays a major contributor role in accelerating the recovery of ecosystem structure and functioning (i.e., supporting the soil traits, land management and topography, climate patterns) and also supports trees' growth and development processes (Gorman et al. 2001; Morrison and Lindell 2011; Rawat et al. 2020). Although the vegetation reestablishment or reforestation treatments after mining have tended to recover degraded forest lands, forests show strongly different habitats in reforested sites than natural forests (Brockerhoff et al. 2008; Bi et al. 2010; Baldrian 2017) because they are generally constituted by mono‐specific trees and can be more likely considered to be a “quick fix” process (Noble and Dirzo 1997). In general, natural forests provide productive habitat and specific ecosystem benefits for native forest species which are high wood production, soil structure supports microbiome's life, biodiversity richness, organic matter, and nutrients (Brockerhoff et al. 2008). Previous studies indicated that the sparse environmental condition limits tree growth (Davison and Jefferies 1966; Williams et al. 1990). Trees have thus differences in their growth and development between reforested and natural forests. In reforested forests, trees do not grow and develop sufficiently due to the low nutrient availability (higher C/N ratio) and contrast matrix (Hirzel et al. 2012).

However, there is no adequate information related to how trees grow and develop between postmine land restoration sites and natural forests. In this study, we therefore aimed to evaluate the success of reforestation practices of mine land restoration. In this context, the morphological, anatomical, and soil traits of *P. nigra* trees between mine land restoration and natural forest sites were analyzed. We hypothesize that (i) the growth and development of *P. nigra* trees are mainly driven by forest structure (reforested or natural) and soil properties; (ii) the wood anatomical properties of *P. nigra* trees are not directly affected by forest structure (reforested or natural); (iii) while the soil properties of *P. nigra* trees are governed by the forest structure (reforested or natural). Understanding the soil traits, growth and develeopment of *P. nigra* reforestation in mine land restoration sites will ensure the success of future plantations.

## 2 Materials and Methods

### 2.1 Study Area

**FIGURE 1:**
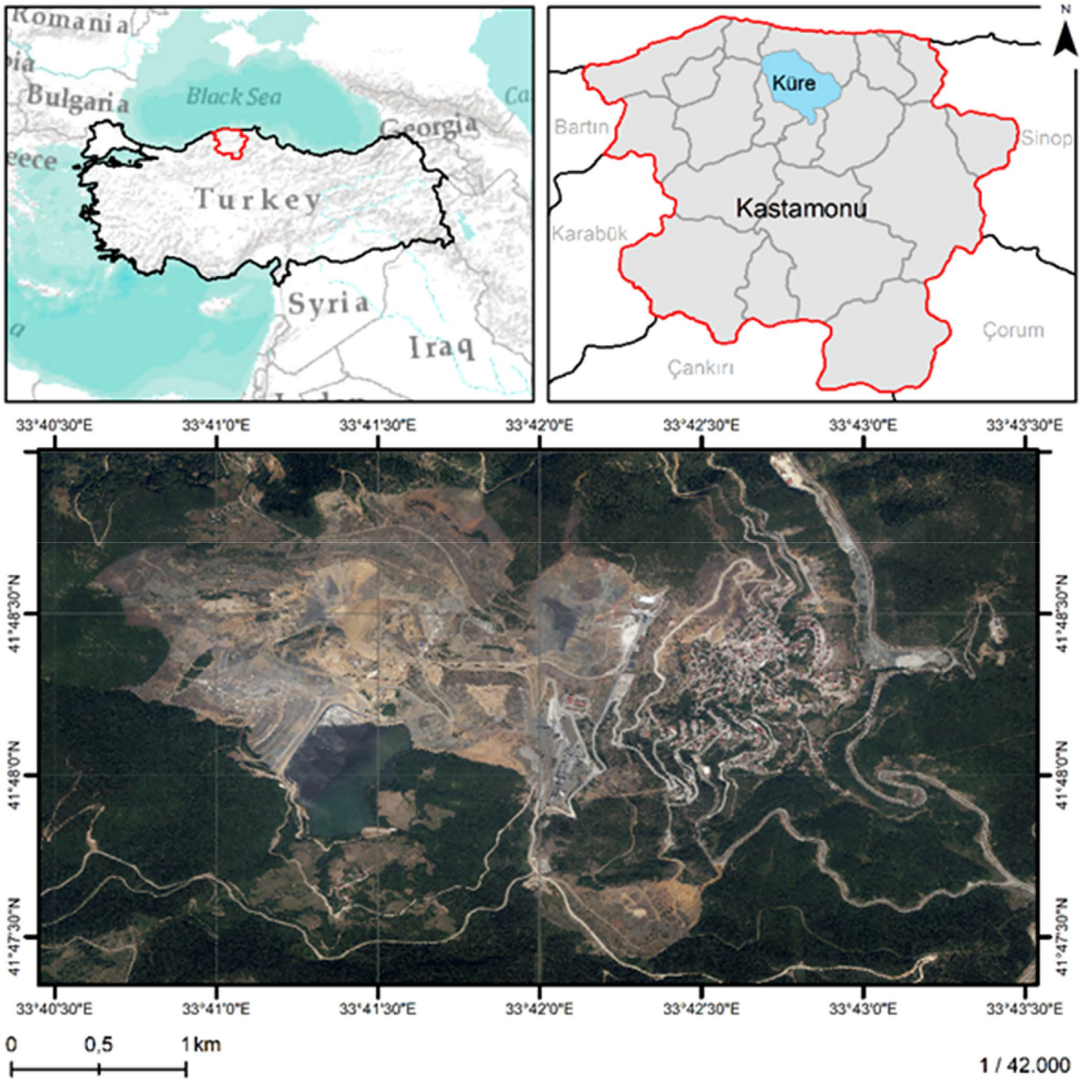
The location of study site in Kastamonu, Türkiye.

This study was conducted at Küre Kastamonu Copper Mine, which is 56 km north of Kastamonu, Türkiye (Figure 1). In the mining site, the ecological restoration and recreation of open‐pit mining areas such as greening of open‐pit mining areas and land reclamation were first started in 2009 within the scope of the aim of restoring forest areas to nature.

**FIGURE 2:**
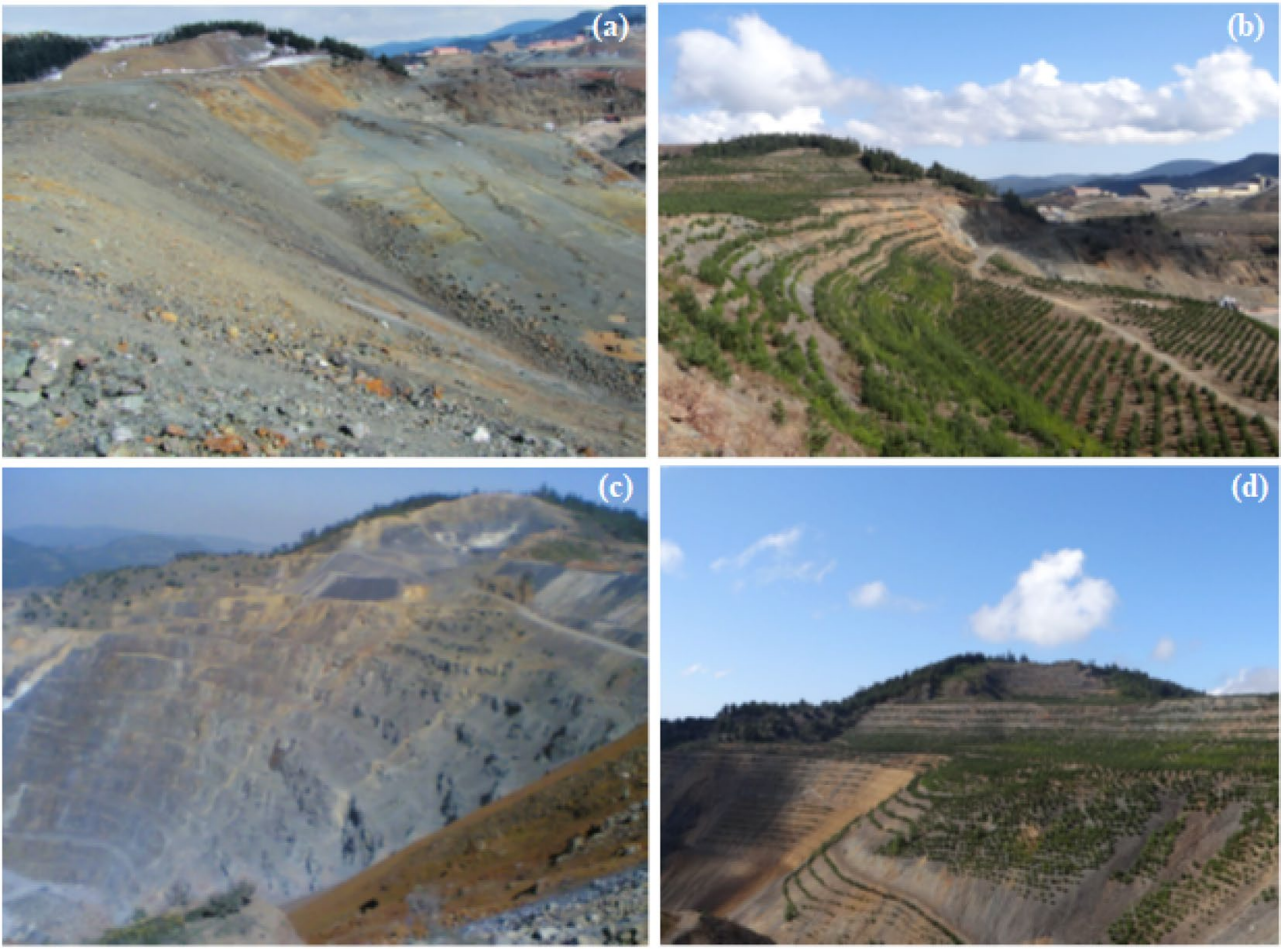
The restoration of mining area. (a, c) the mining area prior to afforestation (b, d) the mining area after afforestation.

The mining company carried of reforestation studies in 300 ha area after mine closure and *P. nigra* was a commonly used species in the plantations. Prior to the mining, the natural forest was dominated by *Pinus nigra* Arnold., Trojan fir (*Abies nordmanniana* subsp. *equi‐trojani*), Oriental beech (*Fagus orientalis* L.), and European hornbeam ( *Carpinus betulus* L.). In 2009, a total of 600 five‐year‐old seedlings of *P. nigra* were planted in mining areas to facilitate the reclamation of degraded lands (Figure 2).

This study was conducted in both postmine land restoration sites and natural forests to determine the restoration success of reforestation. Mine land restoration site and the natural *P. nigra* forest site had similar altitudes, climatic conditions, and similar stand ages (11 years old). The distance between the two sites was approximately 8 km. The altitude ranged from 1100 m to 1250 m in two study sites. The study areas are located within the Euro‐Sberian fito geographic region (Çolak et al. 2009), which is represented by the forests that are composed of broad‐leaf and needle‐leaf trees. The area has a terrestrial Black Sea climate with cold winters and rainy summers. This study was conducted in 2022. The mean annual precipitation was approximately 1072 mm. The annual mean temperature is approximately 4.9°C, and the minimum and maximum monthly means are −7.2°C (coldest month) and 16.6°C (warmest month). In the study site, brown forest soil types are common.

### 2.2 Tree Sampling and Stem Stand Characteristic Measurements

In 2022, the morphological characteristics were measured in stand *P. nigra* trees for each study plot. Total tree heights and diameters at the stem base and breast height (DBH), annual shoot elongation, Height‐to‐crown base (HCB, m), and crown width were measured for all trees in each study site. The height for each tree was estimated with a 3‐m pole with height markings at 0.25‐m increments. The diameter at the stem base was obtained at approximately 2.5 cm above the ground surface using calipers. The diameter at breast height (DBH) was measured using also a caliper. The annual shoot elongation was determined by measuring the distance between terminal bud scale scars (internodes or branch whorls) along the main stems. The growth rate (mean length increment cm year^−1^) of trees was then measured as the change in size between measurements, divided by the number of years between measurements (Forrester 2021).

For crown cover, measurements of the widest and shortest area of crown spread were made using a tape measure, and the mean crown was recorded. Height‐to‐crown base (HCB, m) was determined as the length along the main stem of a tree from the bottom of the tree to the height of the live crown base. The crown ratio was calculated as the crown length divided by the total height of the tree (Allensworth et al. 2021).

### 2.3 Tree‐Ring Width and Anatomical Measurements

To determine tree‐ring width (TRW), each tree stand was cut transversely at the breast height of the stem and then the cross‐section discs were obtained. Prior to the TRW measurements, the stem discs were air‐dried in the laboratory and sanded using a belt sander in order to provide clearly distinguishable tree rings. TRW measurements were thus carried out on the well‐prepared surface of sampled discs. The sanded discs were cut into small pieces for wood anatomical analyses. The small dry pieces were softened in boiling water for approximately 1 day then the specimens were boiled in a mixture of water, glycerol, and ethanol in volume proportions 1:1:1. The softened wood pieces were then microsectioned in transverse and radial directions. Transversal and radial sections were prepared at almost 15 μ in thickness. The thin sections were stained with a solution of 1% safranin for 3–5 min (Bond et al. 2008; Yaltırık 1971). For the wood anatomical analyses, tracheid length (TL), tracheid width (TW), tracheid diameter (TD), tracheid lumen diameter (TLD), tracheid lumen area (TLA), tracheid wall thickness (TWT), ray height (RH), and ray width (RW) were measured. The TL and TW size measurements were conducted using Franklin's (1945) method. The softened wood pieces were first cut into almost 1 × 10 mm small strips and then macerated in a mixture of hydrogen peroxide and concentrated glacial acetic acid in volume proportions 1:1. In order to capture wood anatomical traits, the Leica DM750 light microscope (Leica Microsystems Ltd., Switzerland) with Leica Application Suite (LAS EZ) Image Analysis Software (version 3.4.0. 2016) was used. The IAWA List of Microscopic Features for Softwood Identification method was used in wood anatomical analyses (IAWA Committee 2004).

### 2.4 Soil Sampling and Analysis

A total of 100 soil samples were obtained in 2022. The soil samples were taken from the same area where *P. nigra* trees were harvested (mine land restoration site and forest site). For soil sampling, we used a soil steel cylinder with a height of 5 cm, and soil cores were collected from a maximum depth of 20 cm from the study sites (Gent Jr et al. 1983; Kozlowski 1999; Özden Keleş and Savaci 2021). In the study area, the soil samples were extracted on a regular grid of points approximately every 3 m with each tree growing site. At three random locations within each plot, soil cores were collected and these three samples were composited into one sample for each plot. Therefore, 20 soil cores were composited for each block and each site had four soil samples for analysis. We collected almost 500 g of soil cores from each sample, then the soil cores were placed in plastic bags to determine soil properties. The bulk density of soil cores was calculated as the dry weight of cores divided by their volume (Blake and Hartge 1986). For each study plot's samples, 10 g soil cores were prepared and oven‐dried at 105°C for 24 h to investigate soil moisture content. The mass of oven‐dried samples was then measured. The Loss‐on‐Ignition procedure was used to determine organic matter content. Soil samples were sieved into coarse and fine fractions (2‐mm sieve). The fine soil fraction (≤ 2 mm) was analyzed for chemical properties including soil pH and soluble salts (as electrical conductivity). For each soil core, the nutrient concentrations were determined which are calcium (Ca), magnesium (Mg), phosphorus (P), potassium (K), iron (Fe), copper (Cu), zinc (Zn), manganese (Mn), molybdenum (Mo), selenium (Se), and boron (B) using an energy dispersive X‐ray Fluorescence Spectrometer (EDXRF Xepos II(R) (2016), SPECTRO‐Analytical Instruments GmbH′ Kleve Germany).

### 2.5 Statistical Analysis

The effect of study sites (reforested mine land site and forest site) on tree stem height, DBH, tree growth rates, tree‐ring widths, anatomical features (tracheid length and width, tracheid lumen width, tracheid wall thickness, ray height and width) and soil traits was tested using one‐way analysis of variance. Probability levels *p* < 0.001 and *p* < 0.05 were considered to show a significant relationship. Statistical tests were performed using the SPSS software (Version 23.0).

## 3 Results

### 3.1 Stem Stand Characteristics

**FIGURE 3:**
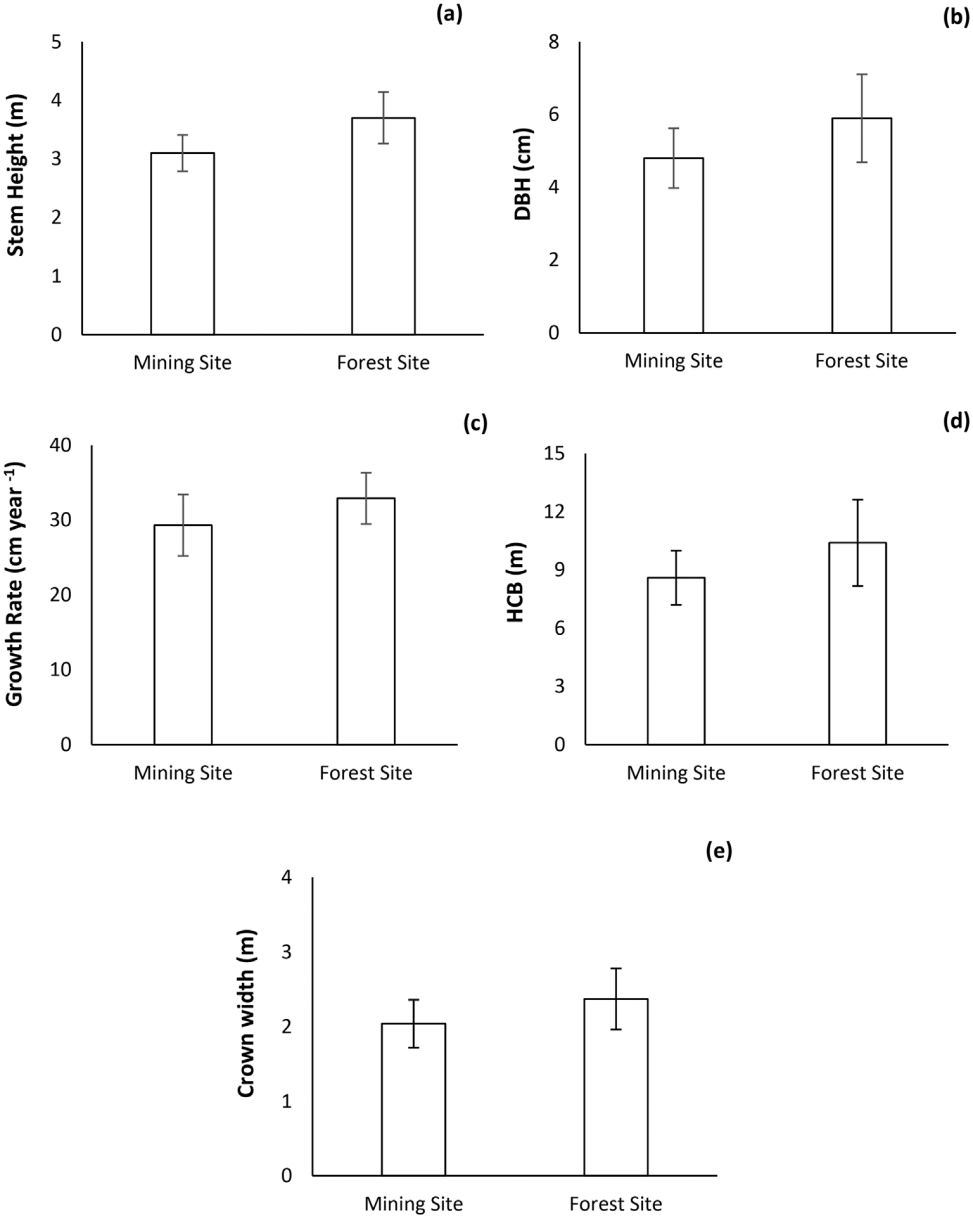
Study plot variables and summary statistics of data between mine land restoration site and *P. nigra* forest site. (a) Total stem heights (b) Diameter at breast height (DBH, cm), (c) Growth rate (cm year^−1^), (d) Height‐to‐crown base (HCB, m), (e) Crown width (m).

The stand characteristics and anatomical properties showed great differences in reforested mine land restoration sites and natural *P. nigra* forests. The total stem height differed significantly between the two study sites (*p* < 0.001). In the natural *P. nigra* forest site, the mean stem height was 3.7 m, and was 3.1 m in the mine land restoration site (Figure 3a). The mine land restoration site and *P. nigra* forest site also exhibited significant differences in DBH values (*p* < 0.01) (Figure 3b). The mean DBH values were greater in *P. nigra* trees grown at the forest site than in *P. nigra* trees grown at the mine land restoration site (mean of 5.9 cm in forest trees and 4.8 cm in the mine land restoration site).

Tree growth rates varied markedly between the two study sites (*p* < 0.01). Tree growth rate across all studied stems ranged from 5 cm year^−1^ to 50 cm year ^−1^. The mean growth rate was slightly greater in the natural forest site than in the reforested mine‐land site; the mean growth rate of the natural *P. nigra* forest was 32.9 cm year^−1^ and the mean growth rate of the reforested mine‐land site was 29.3 cm year^−1^ (Figure 3c).

The mean height‐to‐crown base (HCB) and crown width differed significantly between reforested mine‐land site and forest site (*p* < 0.05). HCB in *P. nigra* forest grown in forest site was 1.2 times higher than in mine land restoration site (mean 10.4 m in forest site and 8.6 m in reforested mine‐land site) (Figure 3d). Crown width slightly varied between the two study sites; mean crown width was slightly larger in natural forest site (2.37 m) than reforested mine‐land site (2.07 m) (Figure 3e).

### 3.2 Wood Anatomical Traits

**FIGURE 4:**
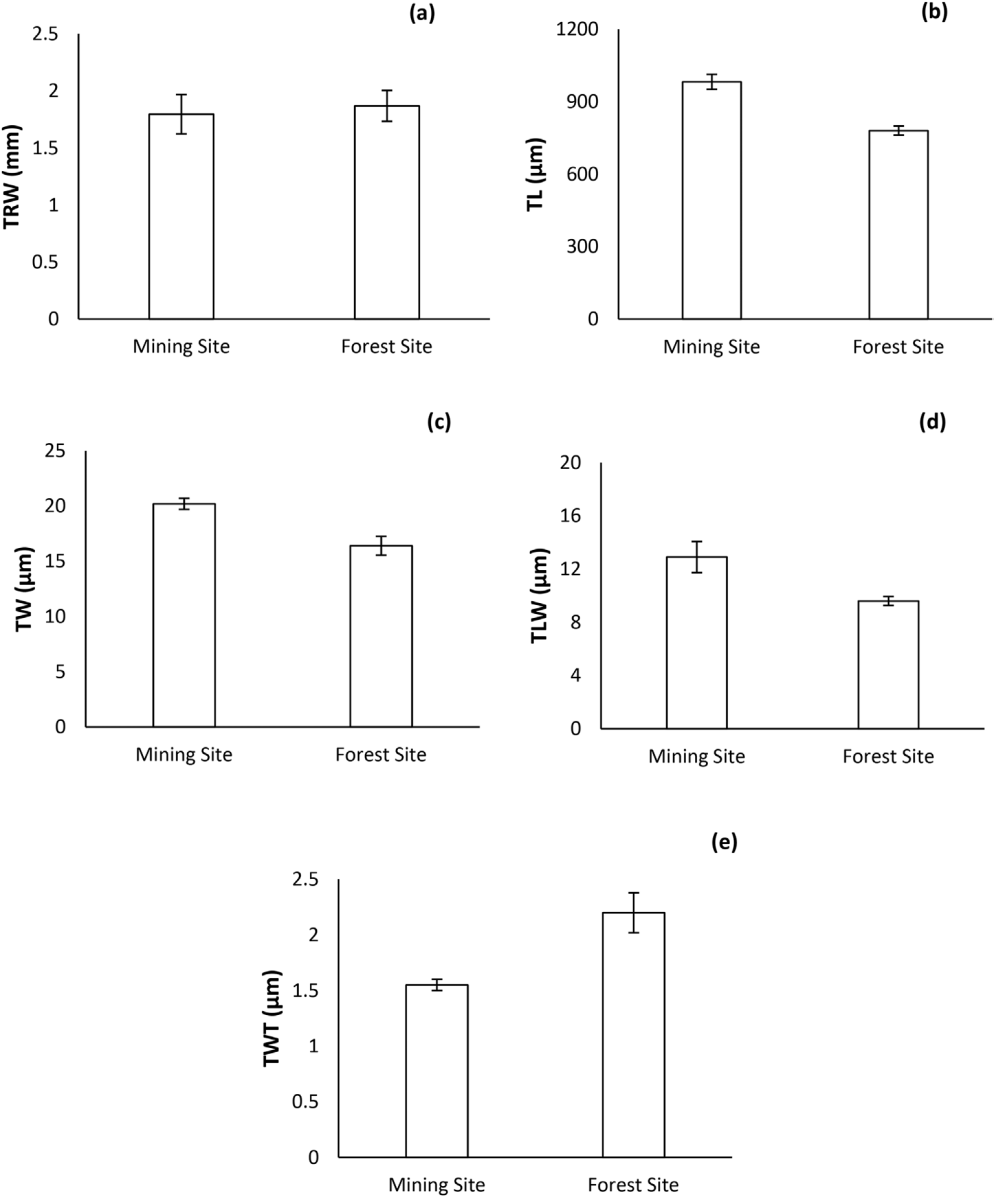
Wood anatomical variables and summary statistics of data between mine land restoration site and forest site. (a) TRW—tree ring width (b) Tracheid length—TL, (c) Tracheid width—TW, (d) Tracheid lumen width—TLW, (e) Tracheid wall thickness—TWT.

The anatomical properties presented significant differences between reforested mine‐land site and the natural forest site (*p* > 0.05). Figure 4 shows the variation in the wood anatomical characteristics of *P. nigra* trees between the two study sites. The captured tree‐ring widths differed between the two study sites (Figure 4a). The mean tree‐ring width was slightly larger in the forest site (1.8 mm) than reforested mine‐land site (1.7 mm), but this difference was not statistically significant between two study sites (*p* > 0.05) (Figure 4a). The tracheid traits also showed different results in two study sites (Figure 4b–e). *P. nigra* trees grown at mine‐land restoration site showed greater TL (982.2 μm), TW (20.2 μm), and TLW (12.9 μm) (Figure 4b–d). However, the difference in mean tracheid lengths (TL) and tracheid widths (TW) did not vary significantly between the two study sites (*p* > 0.05). On the contrary, mean TWT was significantly greater in *P. nigra* trees grown at forest site (2.2 μm) than in *P. nigra* trees grown at mine land restoration site (1.5 μm) (*p* < 0.01) (Figure 4e).

### 3.3 Soil Traits

The measured mean soil physical and chemical features of the study sites are shown in Table 1. The mean soil bulk density and soil chemical traits differed significantly between the two study sites (*p* < 0.05). The reforested mine‐land site had a greater soil bulk density (mean 1.45 g cm^−3^) than in the forest site (mean 1.20 g cm^−3^) The mean total N concentration presented significant variation between the two study sites (*p* < 0.05). The mean total N concentration was on average 1.2 times greater in the forest site (mean 31.2%) than in the reforested mine‐land site (mean 25%).

**TABLE 1:** Mean soil bulk density (g cm^−3^), soil nitrogen (N) content (%), and soil chemical elements of *P. nigra* trees from mine land restoration and forest sites.

Soil sites/Variables	Mine land restoration site	Forest site
Bulk density (g cm^−3^)	1.45 ± 0.04**	1.22 ± 0.06
N content (%)	25 ± 2.4	31.2 ± 8.9*
B (mg kg^−1^)	13760.4 ± 1912.3	13850.8 ± 3644.7^ns^
Se (mg kg^−1^)	42,012 ± 2527.2***	26231.4 ± 4220.9
Mo (mg kg^−1^)	9706.5 ± 531.1***	6565.9 ± 962.7
Ca (mg kg^−1^)	6556.4 ± 828.8 ns	5891.6 ± 1173
Mg (mg kg^−1^)	2809.7 ± 67.4	2942.2 ± 10.2ns
K (mg kg^−1^)	5038 ± 746.6	10332.1 ± 1653.2***
Mn (mg kg^−1^)	519.9 ± 35.1	828.7 ± 240.1 ^ns^
Fe (mg kg^−1^)	45355.3 ± 2216.1***	23424.2 ± 2198.2
Zn (mg kg^−1^)	122.5 ± 9.7***	63 ± 13.7
P (mg kg^−1^)	139.8 ± 20.8	276.9 ± 49.2***
Cu (mg kg^−1^)	465.8 ± 61.2***	154.7 ± 41.2

*Note:* Values indicate the mean ± standart error (SE). The significance is shown as *p < 0.05, **p < 0.01, ***p < 0.001, and ns, not significant.

Abbreviations: B, Boron; Ca, Calcium; Cu, Copper; Fe, Iron; K, Potassium; Mg, Magnesium; Mn, Manganese; Mo, Molybdenum; P, Phosphorus; Se, Selenium; Zn, Zinc.

Overall, *P. nigra* trees grown at the mine‐land restoration site had the highest concentrations of Se (42,012 mg kg^−1^), Mo (9706.5 mg kg^−1^), Ca (6556.4 mg kg^−1^), Fe (45355.3 mg kg^−1^), Zn (122.5 mg kg^−1^), and Cu (465.8 mg kg^−1^) (*p* < 0.001) (Table 1). However, the concentrations of K (10332.1 mg kg^−1^) and P (276.9 mg kg^−1^) were significantly greater in *P. nigra* trees grown at natural forest sites than (*p* < 0.001). The concentrations of B, Ca, Mg, and Mn did not vary significantly between the two study sites (*p* > 0.05) (Table 1).

## 4 Discussion

The world's mineral and energy sources are mainly located in forested areas, and most mine sites are located in forested areas. Mining and mineral processing both are the key drivers of deforestation, degradation of forest cover, loss of land or soil productivity, and biological diversity (Sheoran et al. 2010; Jing et al. 2020; Sonter et al. 2017; Yang et al. 2018; Kayet et al. 2021). However, there is no adequate information related to how trees grow and develop between reforested mine‐land sites and natural forests. In the present study, we therefore compared the growth and development of *P. nigra* trees between reforested postmine sites and natural forests.

In our study site, excavations were made in the mining areas and the openings were filled in depending on its origin. If the filling material is insufficient, gentle slopes were created as much as possible, with gradual transitions compatible with the general view, and in the future, vegetation and trees will be created. The *P. nigra* seedlings have been used for the reforestation which is the site‐specific tree species in the study site. *P. nigra* trees grown at reforested postmine sites and natural forests indicated great differences in their morphological, anatomical, and soil traits. In terms of restoration success, our results showed that *P. nigra* trees grown at natural forests had taller and thicker stems than reforested postmine sites. The growth rate of *P. nigra* trees was also greater in natural forest sites than in reforested postmine sites. The variation in the growth of trees between two sites could be related to the complex biological activity of tree growth because it is affected by many factors such as competition between trees, genetic knowledge and type of individuals, environmental factors and the stage of development (Picard et al. 2012; Borišev et al. 2018). In mining activities, all trees and massive planting of saplings are harvested from the site. When the mining activities are completed, chemical fertilizers are applied to improve soil health status and accumulate tree growth. The litter bacterial community is a primary source of soil organic matter (i.e., N and C cycling) which supports tree growth and development (Bothwell et al. 2014; Laclau et al. 2010). However, the litter bacterial community is strongly dependent on the variations in silvicultural management practices (Yang et al. 2005; Bini et al. 2013) and thus major differences occur in soil properties between reforested sites and natural forests. Previous studies have shown that natural forests tend to have more phylum‐rich soil compared to reforested soils and thus reforested sites have poor soil quality and functions due to the low organic matter content and structure (Behera and Sahani 2003; Gibson et al. 2011; Yang et al. 2005). We found that soil bulk density, soil Se, Mo, Ca, Fe, Zn, and Cu concentrations increased in reforested postmine sites, but soil N, B, Mg, K, Mn, and P concentrations were higher in natural *P. nigra* forests. Our results were consistent with many previous studies, in which plantations had higher soil bulk density and lower soil C and N concentrations than natural forests (Aborisade and Aweto 1990; Solomon et al. 2002; Lemma et al. 2006; Nsabimana et al. 2008). Our findings were also in good agreement with the results from Riva et al. (2023) who studied microbial community and enzyme activity in the soils between reforested sites and natural forests. They found that N, P, Mn, and B concentrations were higher in natural forests than in young plantations. The difference between reforested postmine sites and natural forests may be mainly related to the site preparation, silvicultural activities, and biogeographic conditions (Baule and Fricker 1970; Frouz et al. 2009; Ochal et al. 2010; Pietrzykowski et al. 2013) because abandoned mine soils contain low P, N, K (the primary macronutrients) concentrations which cannot be fixed from the atmosphere by trees therefore the decreased macronutrients and organic matters can limit increment of stand biomass and productivity, as well as root growth of trees in plantations (Forrester et al. 2004; Laclau et al. 2008). We may therefore suggest that particularly the increase in N, P, and K in the soil of natural *P. nigra* forests can provide higher productivity and morphological traits than in reforested postmine sites.

In this study, we also compared the wood anatomical adaptation of *P. nigra* trees between reforested mine‐land site and natural forests. Wood anatomical traits play an essential role in understanding the ecological and adaptive responses of trees to the effect of stressors. Tracheid cells are the main drivers regarding mechanical stability, water transport, and hydraulic safety in conifers (Kramer and Kozlowski 1960; Zimmermann 1983; McDowell et al. 2008). However, tracheids are vulnerable to environmental conditions such that the shape, number, percentage, and size of tracheids alter depending on climatic gradients (Sperry et al. 2006; Castagneri et al. 2017). The silvicultural practices (i.e., selection, thinning, and regeneration) and forest types (plantations or natural forests) have a direct impact on wood anatomical traits by regulating the growth conditions of trees (Mörling 2002; Sperry et al. 2006). In this study, tree rings were slightly wider and tracheid wall thickness was higher in natural forests. Tracheids were longer and wider in reforested postmine sites than in natural forests. However, tree‐ring widths and tracheid traits did not show significant differences between reforested postmine sites and natural *P. nigra* forests. The increased tracheid length and width in plantations relative to natural forests were in good agreement with a previous study by Kara et al. (2021). They investigated the development and anatomical traits of *P. nigra* trees between abandoned agricultural land and forested areas. Tracheids were longer and wider in forested areas, but no significant differences were found in tracheid traits between abandoned agricultural land and forested areas (Kara et al. 2021). No difference between the two study sites could be related to tree‐plantation‐related activities and rehabilitation of degraded lands. In our study site, the topsoil was seriously damaged during mining activities (extraction). However, the mining company carried out the reclamation and rehabilitation activities on the degraded sites to return the land as closely as possible to its original baseline condition after mining activities. Before reforestation in degraded land, topsoil was shaped by spreading evenly on the landscape land until a 15–20 cm top cover was formed. To establish and maintain *P. nigra* plantations, ecological restoration (i.e., vegetable soil laying), mine reclamation, and fertilizer element applications were conducted. Anatomically, the *P. nigra* trees may accumulate water resources and soil mineral nutrients much more readily in reforested postmine sites and tracheid traits can therefore show better properties. We may thus conclude that *P. nigra* trees are anatomically well adapted to mine land restoration sites.

## 5 Conclusion

This study investigated the success of *P. nigra* reforestation after mine closure. We therefore evaluated the variations in morphological, anatomical, and soil traits of *P. nigra* trees between reforested postmine sites and natural forest sites. This is also the first study that investigated the anatomical variations in *P. nigra* trees between reforested postmine sites and natural forests. We analyzed the shoot elongation growth rates of reforested postmine sites and natural forests. Natural forests exhibited greater shoot elongation growth rates than in reforested postmine sites. The total stem height and stem diameters also showed significant differences between the two study sites. *P. nigra* trees had taller and thicker stems in natural forests than in reforested postmine sites. *P. nigra* trees grown at mine land restoration sites and natural forests presented similar tree‐ring widths. However, anatomical traits exhibited quite different results. Although *P. nigra* trees grown at mine land restoration sites had greater tracheid traits (TL and TW), the difference did not vary significantly between two study sites. Overall, *P. nigra* trees grown at the mine‐land restoration site had the highest concentrations of Se, Mo, Ca, Fe, Zn, and Cu. However, the concentrations of N, K, and P were significantly higher in *P. nigra* trees grown at forest sites than in *P. nigra* trees grown at mine land restoration sites. In this study, it is clear that *P. nigra* trees grown in natural forests had greater morphological and soil traits than in mine land restoration sites. However, *P. nigra* trees are anatomically well adapted to mine land restoration sites. Evaluating the morphological, anatomical, and soil traits between mine land restoration and natural forests would play an essential role in the development and establishment of trees. On the other hand, understanding the morphological, anatomical, and soil properties of trees will provide great insights into the success of *P. nigra* plantations in postmining restoration sites.

## Author Contributions

**Seray Özden Keleş:** conceptualization (lead), data curation (lead), formal analysis (equal), funding acquisition (equal), investigation (lead), methodology (equal), project administration (equal), resources (equal), supervision (equal), validation (equal), visualization (equal), writing – original draft (lead), writing – review and editing (equal). **Osman Topaçoğlu:** conceptualization (supporting), funding acquisition (equal), investigation (equal), methodology (equal), resources (equal), visualization (equal), writing – review and editing (equal).

## Conflicts of Interest

The authors declare no conflicts of interest.

## Supporting information


**Data S1:** ece373109‐sup‐0001‐DataS1.xlsx.

## Data Availability

All the required data are uploaded as Supporting Information.
